# Production and Robustness of a Cacao Agroecosystem: Effects of Two Contrasting Types of Management Strategies

**DOI:** 10.1371/journal.pone.0080352

**Published:** 2013-12-02

**Authors:** Rodolphe Sabatier, Kerstin Wiegand, Katrin Meyer

**Affiliations:** 1 Department of Ecosystem Modelling, Büsgen-Institute, Georg-August-University of Göttingen, Göttingen, Germany; University of California, Berkeley, United States of America

## Abstract

Ecological intensification, i.e. relying on ecological processes to replace chemical inputs, is often presented as the ideal alternative to conventional farming based on an intensive use of chemicals. It is said to both maintain high yield and provide more robustness to the agroecosystem. However few studies compared the two types of management with respect to their consequences for production and robustness toward perturbation.

In this study our aim is to assess productive performance and robustness toward diverse perturbations of a Cacao agroecosystem managed with two contrasting groups of strategies: one group of strategies relying on a high level of pesticides and a second relying on low levels of pesticides. We conducted this study using a dynamical model of a Cacao agroecosystem that includes Cacao production dynamics, and dynamics of three insects: a pest (the Cacao Pod Borer, *Conopomorpha cramerella*) and two characteristic but unspecified beneficial insects (a pollinator of Cacao and a parasitoid of the Cacao Pod Borer). Our results showed two opposite behaviors of the Cacao agroecosystem depending on its management, i.e. an agroecosystem relying on a high input of pesticides and showing low ecosystem functioning and an agroecosystem with low inputs, relying on a high functioning of the ecosystem. From the production point of view, no type of management clearly outclassed the other and their ranking depended on the type of pesticide used. From the robustness point of view, the two types of managements performed differently when subjected to different types of perturbations. Ecologically intensive systems were more robust to pest outbreaks and perturbations related to pesticide characteristics while chemically intensive systems were more robust to Cacao production and management-related perturbation.

## Introduction

New paradigms in agriculture based on ecological intensification such as natural farming [1], agroecology [2], [3], the evergreen revolution [4], or the doubly green revolution [5] are presented as challenging alternatives to more conventional farming relying on a high level of chemical input. They are presented as being more respectful of the environment while ensuring a high level of production. A central idea behind these paradigms is that associated biodiversity is strongly impacted by the use of chemicals in conventional farming [6], [7] while the associated biodiversity could provide a large range of ecosystem services that often have the same effect as the chemical used (e.g. pest regulation, [8]). According to these paradigms, reducing the amount of chemicals used would maintain biodiversity and ecosystem services at a high level and ensure high yields with lower economic costs. Moreover, based on the ecological concept of stability of ecosystems [9], agroecosystems with high levels of biodiversity are considered more robust, stable and resilient toward perturbations. In other words, such systems have the advantages of a high productivity due to the maintenance of high levels of ecosystem services [10] and of a strong capacity to resist to perturbations [11].

The three concepts of resilience, stability and robustness, although related, slightly differ: resilience is “the persistence of relationships within a system and is a measure of the ability of these systems to absorb changes of state variables, driving variables, and parameters, and still persist” [12], stability is “the ability of a system to return to an equilibrium state after a temporary disturbance” [12] and robustness is: “the ability to maintain performances in the face of perturbation and uncertainty” [13]. In this study we consider the issue of robustness that addresses the performance of the system when the perturbation occurs.

The overall production of an agroecosystem is quite easy to quantify but its behavior in face of a perturbation is much more difficult to assess as it strongly depends on the perturbation considered [14].

In this study our aim is to assess productive performance and robustness toward diverse perturbations of an agroecosystem managed with two contrasting strategies. More precisely, we address the following two questions:

1. 1: What is the shape of the relationship between robustness to perturbations and yield of an agroecosystem under a broad range of different management schemes?
2. 2: Where are the management strategies based on ecological processes (hereafter called ecological strategies, ES) and the ones based on pesticide use (hereafter called chemical-based strategies, CBS) located within the range of outcomes found in 1) and which of these two types of strategies performs better with respect to yield and robustness?

To answer these questions, we developed a model based on a case study of the Cacao agroecosystem in Central Sulawesi (Indonesia). The aim of this model was to capture some general patterns of the interactions between an agroecosystem, its environment and its management by the farmer. Hence, the product of this study is not intended for direct application in the field, but rather for informing management decisions on a general basis. To calibrate and parameterize this model, the Cacao case study was chosen as it captures the main above-ground ecosystem services and disservices and therefore represents a wide variety of agroecosystems well. Cacao crops were introduced in South East Asia 200 years ago. First records of Cacao production in Indonesia date back to 1848 (15) but production remained low (<5000 t.year^−1^ until the late 1970's; [15], [16]). Production strongly increased in the last decades to reach more than 800 000 t.year^−1^ in 2010 [16]. In Indonesia, Cacao production is continuous but yield is not constant through the year and shows a main peak in January and in some cases a minor peak six months later. Two main pests impact Cacao production: *Helopeltis theobromae* and the Cacao Pod Borer *Conopomorpha cramerella* [17]. Cacao pollination is done by midges (Ceratopogonidae) and is a limiting factor for production [18].

After briefly describing the model that we used, we determine the relationship between ecosystem functioning and the number of spraying events and define two groups of strategies, ES and CBS. Then we determine the relationship between yield and robustness for the set of all possible management strategies and six types of perturbations. We then look more precisely at the relative positions of the two subsets of management strategies ES and CBS on the production-robustness relationship. Our results show that ecosystem functioning is strongly negatively correlated to the number of spraying events and show how both production and robustness of the two extreme types of strategies depend on both the type of pesticide used and the type of perturbation.

## Materials and Methods

### Model overview

Robustness of agroecosystems is difficult to address in the field due to the high complexity of agroecosystems and their low reproducibility for experimental purposes. In this context, modeling approaches are powerful tools. *In silico* experiments can cope with the complexity and their high level of reproducibility makes them useful frameworks to represent complex agroecosystems and to study their robustness to perturbations.

**Figure 1:**
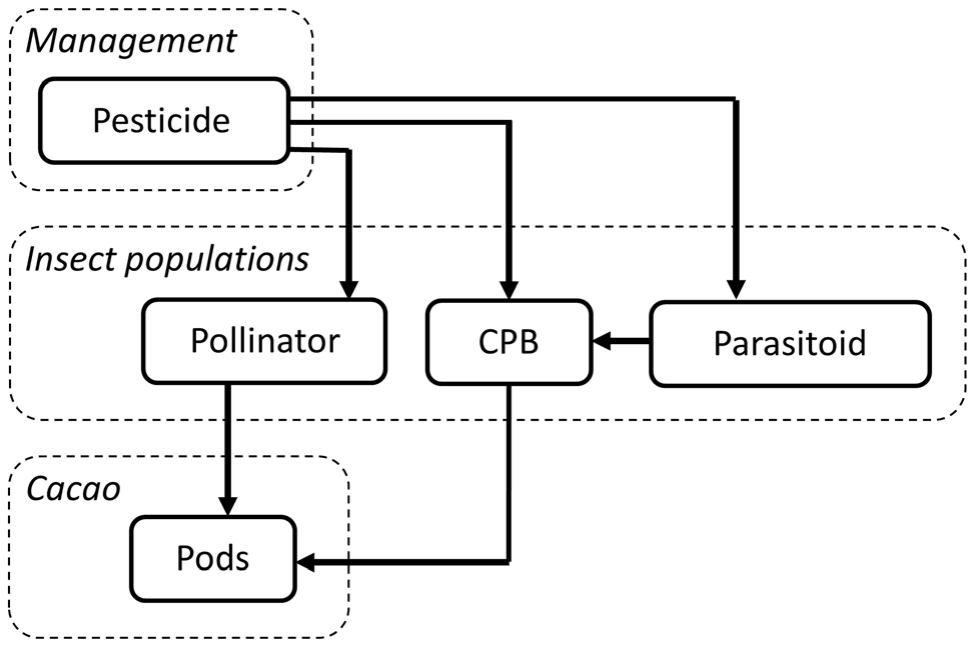
Overview of the model structure. CPB stands for Cacao Pod Borer. Bold black arrows stand for the processes considered in the ecosystem functioning index (EFI; eqn 4).

The study that we present here is based on a model developed and presented in [19]. This model gives an agroecological representation of a Cacao plantation (Figure 1). It is a discrete time model with a time step of one month and a time horizon of 20 years (T = 240 months). It links the Cacao pod dynamics to the population dynamics of a pest species (the Cacao Pod Borer, *Conopomorpha cramerella*) and two characteristic but unspecified beneficial insect populations (a pollinator of Cacao and a parasitoid of the Cacao Pod Borer). The Cacao Pod Borer and parasitoid parts of the model were inspired by both the Cacao model of [20] and the more general Nicholson and Bailey host-parasitoid model [21].

The insects impact the Cacao yield in several ways. The pollinators *N_Pol_* positively affect the number of pods of age 0 *Pods_0_* by pollinating the obligately outcrossing Cacao plants. The Cacao Pod Borer population reduces the amount of Cacao beans eventually harvested. The Cacao Pod Borer population *N_CPB_* is regulated by a parasitoid *N_Par_*. All three insect populations are affected by the use of pesticides by the farmer. We distinguish two effects of spraying: the efficiency *η* (the effect on the Cacao Pod Borer) and its selectivity θ (the ratio of effects on beneficial and on pest populations). Efficiency and selectivity range from 0 to 1, η = *1* means that 100% of the Cacao Pod Borer are killed by the pesticides, θ = *1* means that the effect of pesticide application on beneficial insects is as strong as its effect on the Cacao Pod Borer. Since the aim of pesticide application in the field is to control the Cacao Pod Borer, we limited the study to pesticides having a stronger effect on the target species (Cacao Pod Borer) than on the other species (beneficial insects). We therefore implicitly assume that farmers would not use pesticides that have a net negative impact. In this sense, we avoided the trivial situation where pesticides should be banned due to net negative effects. The model computes the Cacao yield dynamics *Y* through time as well as the dynamics of the three insect populations for different types of pesticides (characterized by their selectivity and efficiency), and for different timings of spraying.

The model can be described by the following system:
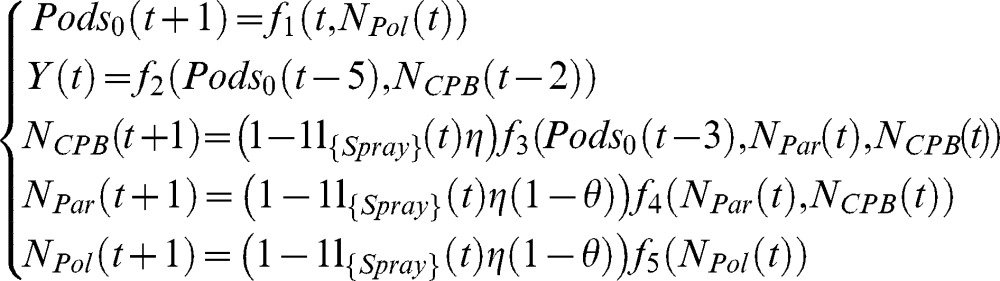
(1)Cacao Pod Borers preferentially attack pods of age 3, pods are harvested at age 5. 1_{*Spray*}_, the characteristic function related to the spraying event is defined as follows:
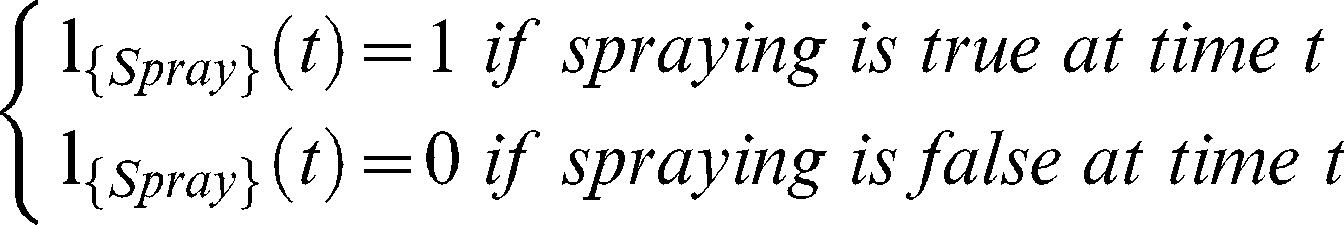
(2)
*f_1_–f_5_* are the functions related to the different natural dynamics (see Appendix S1, eqn S.1.1, S.1.11, S.1.8, S.1.9 and S.1.6)

### Model calibration and sensitivity analysis

Most parameter values were taken from the literature, when possible from studies conducted in central Sulawesi (Table 1); [19].

**Table 1:** List of parameters.

Name	Description	Value	Unit	Reference
Intra-annual dynamic of Cacao pod production (eqn S.1.2)				
*α_1_*	*Mean sinusoid 1*	7.176	pods.ha^−1^	[23] *,[24] *
*β_1_*	*Amplitude sinusoid 1*	1.425	pods.ha^−1^	[23] *,[24] *
*Ω*	*Time period sinusoids*	12	months	[23] *,[24] *
Inter-annual trend of Cacao pod production (eqn S.1.3)				
*α_A_*	*Age dependence parameter 1*	3.82	-	[42]
*β_A_*	*Age dependence parameter 2*	0.086	-	[42]
*γ_A_*	*Age dependence parameter 3*	1.33	-	[42]
*A_0_*	*Initial age*	120	months	[42]
*φ_A_*	*Standardization coefficient*	2.5 10^−3^	-	[42] *
Pollination effect (eqn S.1.4, eqn S.1.5)				
*α_P_*	*Pollination factor 1*	−0.83	kg. ha^−1^.	[18]
*β_P_*	*Pollination factor 2*	0.34	kg. ha.^−1^ pollinated flower^−1^	[18]
*γ_P_*	*Standardization coefficient*	2.36	ha.kg^−1^	[18] *
*α_ψ_*	*Pollinated flowers per pollinator unit*	40	pollinated flower.pollinator^−1^	[18]
Effects of management (eqn S.1.6)				
*η*	*Spraying effect on pests (efficiency)*	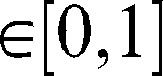	-	-
*θ*	*Spraying effect on beneficial insects (selectivity)*	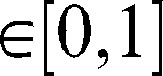	-	-
Pollinator dynamics (eqn S.1.6)				
*λ_P_*	*Growth rate*	100	-	[22] *
*c_P_*	*Competition rate*	99	pollinator^−1^	[22] *
Cacao Pod Borer (CPB) dynamics (eqn S.1.8)				
*α_CPB_*	*Number of eggs per female*	130	egg.female CPB^−1^	[20]
*β_CPB_*	*Egg predation rate*	0.15	-	[20]
*S_0_*	*Larvae survival*	0.1	CPB.egg^−1^	[20] *
*σ_CPB_*	*Adult sex-ratio*	0.5	female CPB. CPB^−1^	Wielgoss & Clough (unpub)
*δ_CPB_*	*Adult predation rate*	0.41	-	[20]
*μ*	*Density dependence coefficient 1*	0.69	pod.CPB−^1^	[20]
*ρ*	*Density dependence coefficient 2*	1.92	-	[20]
Parasitoid dynamics (eqn S.1.9, eqn S.1.10)				
*a_Par_*	*Parasitism probability*	0.003	-	[20] *
Yield function (eqn S.1.12)				
*ω*	*Weight of dry beans per pod*	0.03	kg.pod^−1^	Clough (unpub)*
*α_Y_*	*Yield loss parameter 1*	0.01	CPB.pod^−1^	[20]
*β_Y_*	*Yield loss parameter 2*	6.33	-	[20]

* refers to parameters calibrated from the cited reference.

Data on pollinators of the Cacao (ceratopogonid midges) are very scarce and we could not calibrate this dynamics on data from any of the pollinating species. Therefore we relied on available data from another tropical species of the same family: *Leptoconops albiventris*. Although it concerned a different species than the pollinators of our system, we considered these data suitable enough regarding the ambitions of the model. These data [22] provide a 6-months survey of a population of midges subjected to regular pesticide applications and were used to calibrate the equation corresponding to pollinator dynamics. Pesticide applications are followed by an instantaneous drop of the midge population followed by a fast recovery of the population. Given that we do not aim at quantitative prediction but at qualitative understanding, we only modeled relative abundances and standardized the midge population in the absence of spraying to 1000 at equilibrium.

A sensitivity analysis was then conducted to test the effect of a variation of +/− 10% of each parameter of the model on the average Cacao yield and on the average population sizes of each of the three insects (for details, see [19]). The sensitivity analysis showed a high sensitivity of the model to the two parameters of the intra-annual dynamics of Cacao pod production (parameters *α_1_*, and *β_1_* in appendix S1). Calibration of these key parameters was then adjusted using data from a survey of the Cacao yield of two plots in central Sulawesi [23], [24]. Initially conducted to compare rainfall treatments, this database provided us with six control subplots that we used for calibration. The survey was conducted from January to December 2007 with a two-week time step. Calibration was obtained by minimizing the Root Mean Square Error (RMSE) of the full model compared to the Cacao yield data. To ensure a periodic pattern of the dynamics, calibration was made on two successive years. This sensitivity analysis made it possible to isolate the most sensitive parameters. Once the sensitivity to input data had been tested, all simulations were run in a deterministic manner, to keep the number of simulation within feasible limits.

### Simulations

#### Indices

The model that we used made it possible to compute the agroecosystem dynamics for the whole set of possible management strategies. For each simulated spraying strategy, we recorded the average yearly yield and the number of spraying events as well as two specific indices to record information on robustness and ecological functioning of the system. The Robustness index records the average deviation (in absolute values) of the productive output for a given set of perturbations. It reads as follows:
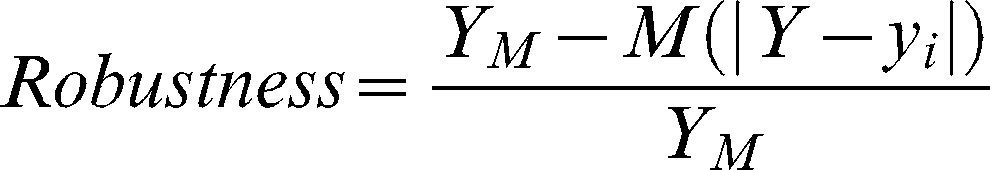
(3)


The Ecosystem Functioning Index (EFI) relates to the ecological functioning of the agroecosystem. It synthesizes the different ecological processes at stake and reads as follows:
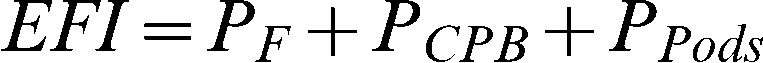
(4)with *P_F_* the pollination rate, *P_CPB_* rate of parasitism of the Cacao Pod Borers and *P_Pods_* the infestation rate of the Cacao pods. This index synthesizes all ecological functions at stake in our modeled agroecosystem and does not only focus on ecosystem services. We decided to consider all functions regardless of their effects on production to reflect information available in real systems. Indeed, in real systems it is difficult to put a number on services specifically, especially when the distinction between services and disservices is not clear [25] or when the services are not known well.

#### Typology of the management strategies

To identify spraying strategies that combine extreme intensities of management and ecosystem functioning we built two contrasting groups of strategies, depending on their position in the ecosystem functioning (*EFI*) - number of spraying events (*N_S_*) - plane. The set of Ecological Strategies (ES) encompass all strategies that are within both the 10% *EFI* upper quantile and the 10% *N_S_* lower quantile. The set of Chemical Based Strategies, i.e. the CBS-management strategies encompass all strategies that are within both the 10% *EFI* lower quantile and the 10% *N_S_* upper quantile.

#### Production and robustness of the management strategies

We analyzed the relationship between yield and robustness of the system under six types of perturbations related to management or environmental conditions. More precisely, we subjected the system to the following perturbations.

Perturbations related to modifications of the environmental conditions.

Pest outbreak: For each management strategy, we simulate two alternative perturbations via a sudden increase in the Cacao Pod Borer population by 500 and 1000 individuals.ha^−1^ at month 3 (time of the year where the number of pods sensitive to this pest is the highest).
Variation in Cacao production: For each management strategy, we simulate four alternative situations with each of the two parameters of the Cacao pod dynamics (mean and amplitude of the pod production sinusoid) increased or decreased by 10%. This perturbation could reflect diverse environmental variations, including climatic ones.

Perturbations related to modifications of the management strategies.

Event shift: For each management strategy, we simulate 12 alternative, modified strategies involving the shift of one of the 12 spraying/non-spraying events (i.e. 1 spraying event was replaced by a non-spraying event and *vice versa*).
Temporal shift: For each management strategy, we simulate two alternative strategies by shifting the entire spraying sequence by one month (either shifted one month earlier or one month later).

Perturbations related to the pesticide characteristics.

Pesticide efficiency: This scenario corresponds to a modification of the pesticide efficiency. For each management strategy, we simulate two alternative situations with a pesticide whose efficiency is either increased or decreased by 20%.
Pesticide selectivity: This scenario corresponds to a modification of the pesticide selectivity. For each management strategy, we simulate two alternative situations with a pesticide whose selectivity is either increased or decreased by 20%.

These six perturbations were tested on the 2^12^ possible management strategies. Then the relative performances of the ES and CBS defined were assessed. To compare the performances of ES and CBS, we compared all possible pairs of strategies (always one from the ES set and one from the CBS set) and computed the proportion of ES that performed better than CBS (hereafter called Comparison Index, *CInd*).

We first give a detailed analysis of the response of the Cacao agroecosystems to these perturbations for a single type of pesticide (Efficiency = 0.5, Selectivity = 0.5). Then, we conduct the same analysis for 81 different pesticide types (Efficiency and Selectivity ranging from 0.1 to 0.9 with a step of 0.1) so as to test the range of validity of these first findings.

Numeric computations and statistical analyses were performed with Python 2.7.2 (http://www.python.org/).

## Results

### Calibration

**Figure 2:**
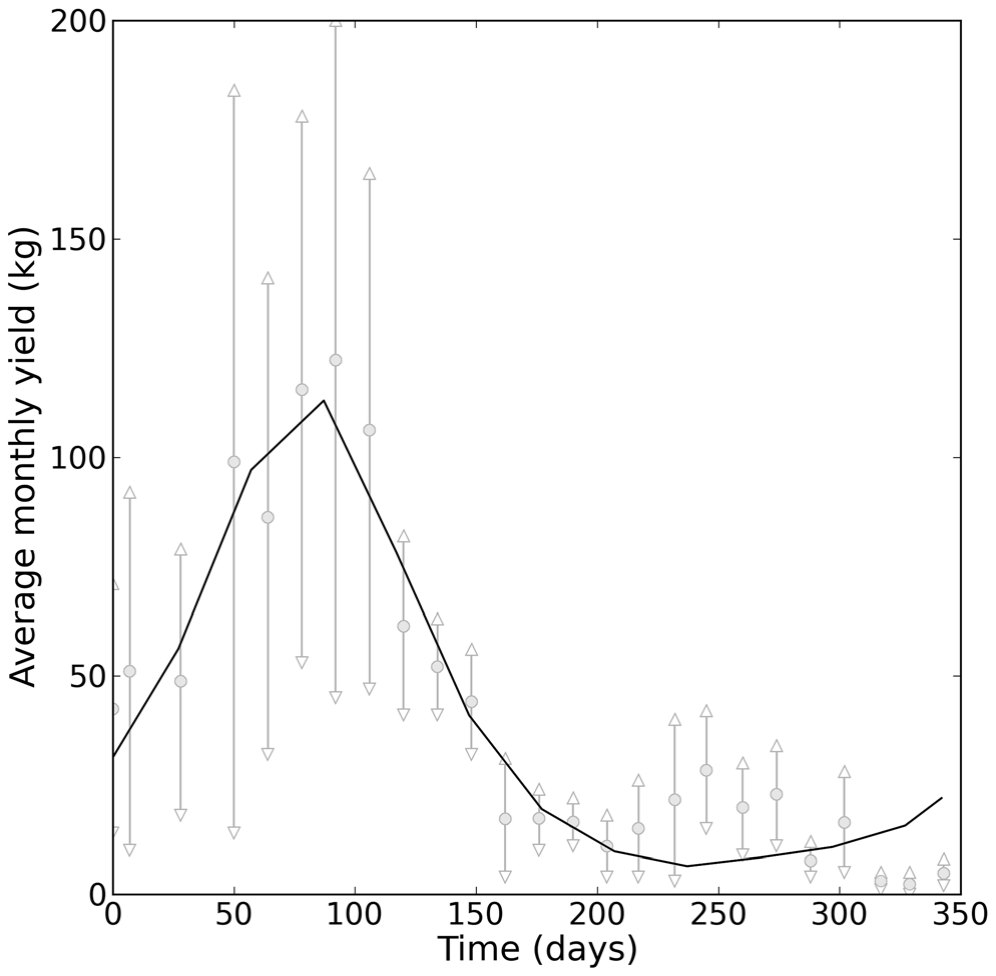
Model output and data from the study area (zoom on year 10). The solid black curve corresponds to the model output, the gray dots to the average monthly yields observed in the six plots of the study area. The grey bars around these dots correspond to the average values +/− standard deviation.

After calibration, our model gave a reasonable visual fit (Figure 2). Comparison of the model outputs with the Cacao yield data showed that we managed to capture the general behavior of the system.

### Typology of the management strategies

**Figure 3:**
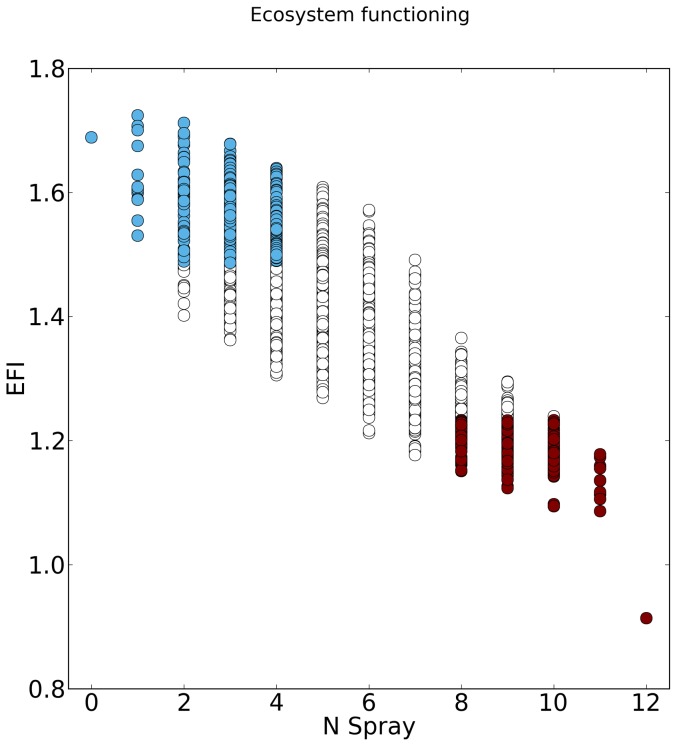
Intensity of spraying and ecosystem functioning of the different spraying strategies. The x-axis is the Ecosystem Functioning Index (EFI) and the y-axis is the number of spraying events per year. Each symbol represents one of the possible spraying strategies. Black symbols stand for the subset of Chemical Based Strategies (CBS; high spraying intensity and a low ecosystem functioning), white symbols stand for the subset of Ecological Strategies (ES; low spraying intensity and a high ecosystem functioning). Gray symbols stand for all other strategies.

We logically observed a strong relationship between the number of spraying events *N_S_* and the functioning of the agroecosystem *EFI* (Figure 3; *EFI = 1.63−0.05 N_S_*, p<10^−3^, R^2^ = 0.68). The management strategies built a continuum. At the two extremes of this continuum, we distinguished two subsets of trajectories: the Chemical Based Strategies (CBS) that corresponded to a high number of spraying events and a low functioning of the ecosystem and the Ecological Strategies (ES) that corresponded to a low number of spraying events and a high ecosystem functioning (Figure 3).

### Production and robustness of the management strategies

The relationship between yield and robustness differed between the different types of perturbations (Table 2). Negative relationships were found for environment- and management-related perturbations while a positive relationship was found for pesticide-related perturbations.

**Table 2:** Relationship between yield and robustness for four types of perturbation (linear models).

Perturbation	Trend	Intercept	Slope	R^2^	P-value
Environment (pest outbreak)	Negative	600	−160	0.03	<10-3
Environment (production)	Negative	1158	−1018	0.65	<10-3
Management (event shift)	Negative	2478	−2053	0.14	<10-3
Management(temporal shift)	Negative	859	−413	0.03	<10-3
Pesticide (selectivity)	Positive	−270	747	0.37	<10-3
Pesticide (efficiency)	Positive	−478	944	0.19	<10-3

**Figure 4:**
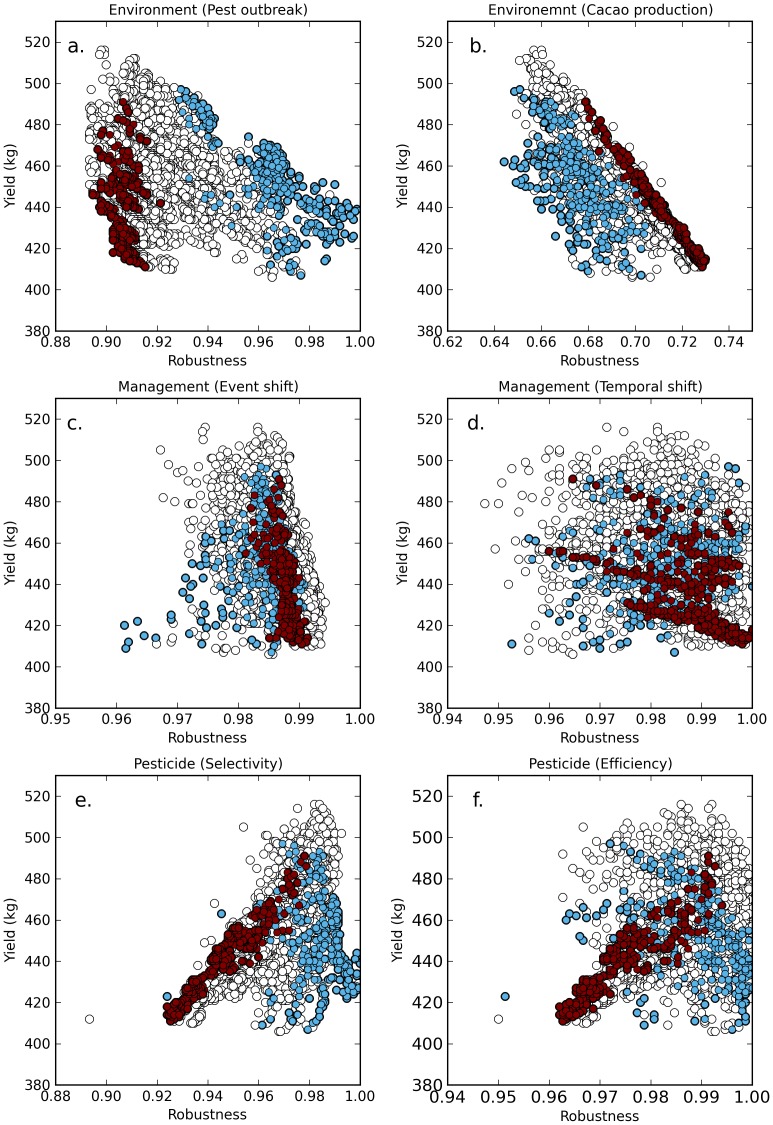
Relationship between yield and robustness under different perturbations. Under environmental perturbation (a: pest outbreak; b: production variation), under a modification of the management strategy (c: shift in one of the spraying/non spraying events; d: temporal shift) or under a perturbation linked to the type of pesticide (e: selectivity; f: efficiency). Each white symbol represents one of the 4096 possible spraying strategies. Dark red symbols stand for the subset of Chemical Based Strategies (CBS, high spraying intensity and a low ecosystem functioning), light blue symbols stand for the subset of Ecological Strategies (ES, low spraying intensity and a high ecosystem functioning).

On average, the yield obtained with ES was higher than the yield obtained with CBS (Figure 4, *CInd* = 0.71). The robustness of CBS was lower than the one of the ES under perturbations due to a pest outbreak (Figure 4.a.; *CInd* = 1.00) as well as under pesticide-related perturbations (Figure 4e, *CInd* = 0.99 and Figure 4f, *CInd* = 0.80). However, robustness of CBS was higher than the robustness of ES under perturbations related to management (Figure 4c, *CInd* = 0.12 and Figure 4d, *CInd* = 0.32) or to production (Figure 4b, *CInd* = 0.02).

These results can be qualitatively explained in the following way. Systems managed with ES have a high level of ecosystem functioning. This gives a higher capacity of self-regulation to the system, which explains its high robustness to pest outbreaks and to the pesticide characteristics. However ES are very specific strategies aimed at “driving” the system instead of “controlling” it. This explains their low robustness to both production- and management-related perturbations.

### Effect of the type of pesticide

**Figure 5:**
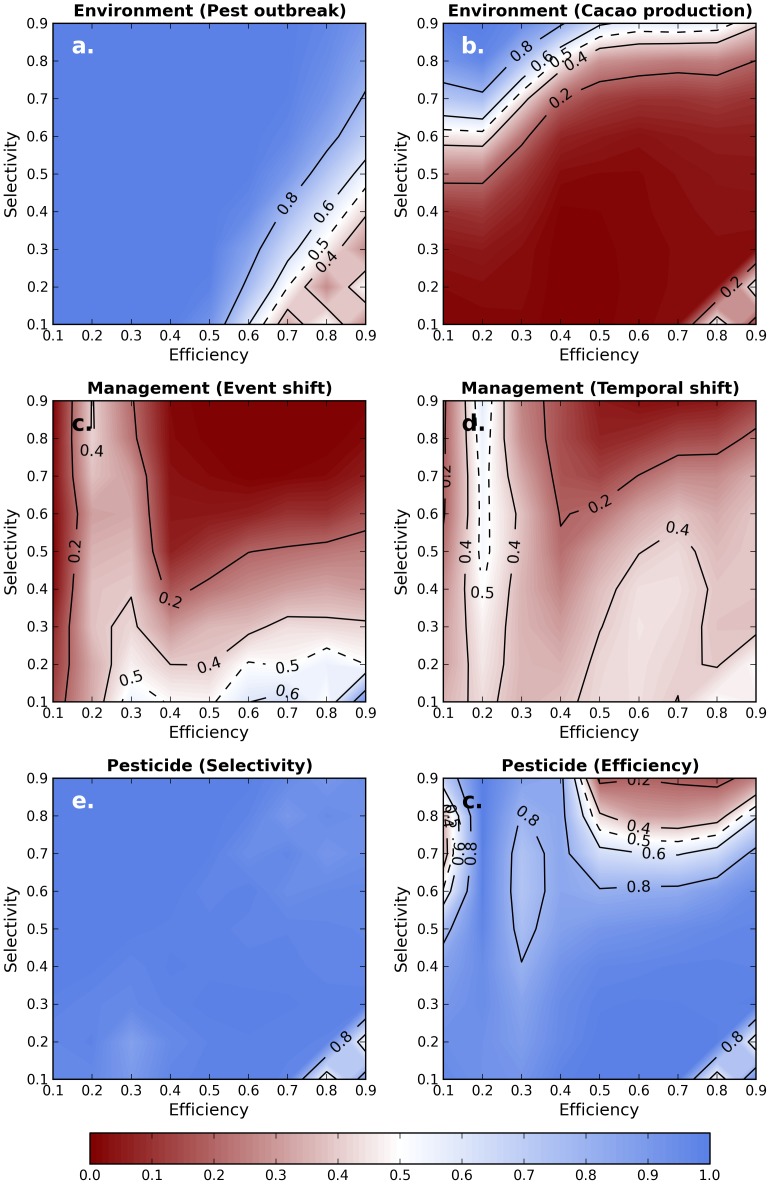
Comparison of the robustness of the agroecosystem managed by Chemical Based Strategies (CBS) or Ecological Strategies (ES) under 6 different perturbations. Figures a and b correspond to an environmental perturbation (a: pest outbreak, b: production perturbation). Figures c and d correspond to perturbation of the management (c: shift of one spraying/non spraying event, d: temporal shift of the management strategy). Figures e and f correspond to perturbations of the pesticide characteristics (e: selectivity; f: efficiency). Color indicates the comparison index calculated, i.e. the percentage of pairs of strategies in which the ES performs better than the CBS.

Comparison of the robustness of CBS and ES showed similar results for the different types of pesticides as for the first pesticide detailed in the former section (Figure 5). Due to the non-linearity of model dynamics, the patterns observed are not necessarily smooth but general conclusions can still be drawn. For most pesticides, CBS were more robust than ES to Cacao production-related perturbations (average *CInd* = 0.22) as well as to management-related perturbations (event shift, average *CInd* = 0.24, temporal shift, average *CInd* = 0.32) and ES were more robust than CBS to pest outbreaks (average *CInd* = 0.88) as well as to pesticide-related perturbations (efficiency, average *CInd* = 0.80, selectivity, average *CInd* = 0.96).

**Figure 6:**
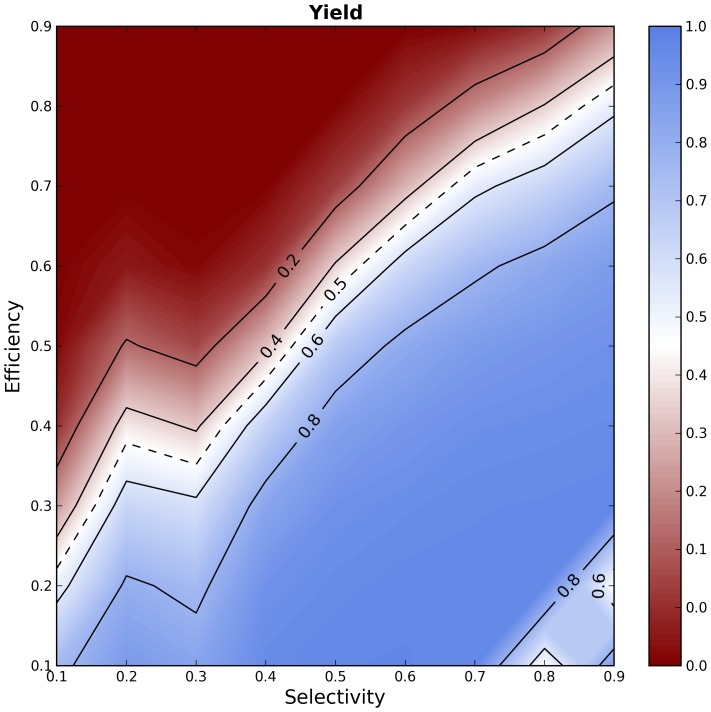
Comparison of the yield obtained in agroecosystems managed by Chemical Based Strategies (CBS) or Ecological Strategies (ES). Color indicates the comparison index calculated, i.e. the percentage of pairs of strategies in which the ES performs better than the CBS.

However, yield showed a more balanced pattern (Figure 6, average *CInd* = 0.51). ES showed higher yields with pesticides of high efficiency and low selectivity while CBS showed higher yields with pesticides of high selectivity and low efficiency. This illustrates the non-trivial role of pesticides in a complex agroecosystem.

## Discussion

Our results showed two opposite behaviors of the Cacao agroecosystem that we modeled depending on their management: First, Cacao agroecosystems relying on a high input of pesticides and showing low ecosystem functioning that fit to the conventional model of chemical intensification inspired by the Green Revolution; second, Cacao agroecosystems relying on ecosystem functioning with a low level of chemical input that fit to the model of ecological intensification inspired by agroecology-like paradigms. These two types of management led to different levels of production as well as different robustness. From the production point of view, no system clearly outclassed the other and their ranking depended on the type of pesticide used. From the robustness point of view, the two types of systems performed differently when subjected to different types of perturbations. Ecologically intensive systems were more robust to pest outbreaks and pesticide-related perturbation while chemically intensive systems were more robust to management perturbation and production-related perturbations.

### Generality of the results

Our results were obtained with a simplified model of a Cacao agroecosystem and the generality of our results should be discussed. The agroecosystem that we modeled only includes three insects, which is far less than what can be found in the Cacao agroecosystem of the study area [26] and in agroecosystems in general (e.g. [27]). However, ecosystem properties depend much more on functional diversity than on species richness *per se* [28] and agroecosystems are no exception [29]. Choosing these three species, we focused on the three main above-ground ecosystem services and disservices commonly found in agroecosystems (pest damage, pest regulation and pollination; [10]). In this sense, even though the quantitative outputs of our models may differ when applied to other agroecosystems, the following qualitative results should remain valid to a broad range of agroecosystems:

There is a strong negative correlation between the frequency of spraying events and the functioning of the agroecosystem.
Higher yields can be reached with farming practices based on ecosystem functioning when broad-spectrum pesticides (high efficiency and low selectivity) are used.
Farming practices based on ecosystem functioning are more robust to pest outbreak perturbations than farming practices based on chemical inputs.
Farming practices based on ecosystem functioning are more sensitive to management perturbation than farming practices based on chemical inputs.

We see two ways of validating these general findings through modelling. These general results could be tested by applying our modeling framework to other types of agroecosystems (e.g. orchards, oil palm plantations,…). The application of other models of agroecosystems (e.g. [30]–[33]) to the question raised in this article would also be a way of testing the generality of these results. However, most of these models would first have to be extended to explicitly include crop dynamics and the effects of insects on production. The use of such models developed in different scientific contexts would contribute to the cross-validation of our results.

### Limits and perspectives

In this article, we only focused on Cacao agroecosystem management at the field scale through the use of pesticides. Therefore, our model could be developed in two main directions to improve our understanding of the mechanisms of ecological intensification: adding new dimensions to management and increasing spatial scale.

With respect to new dimensions, the dynamics of shade trees could be added to the model. In the specific case of agroforestry systems such as Cacao, the management of shade trees is a strong driver of ecosystem services and disservices [34], [35]. Including this aspect in our model would improve its predictive power and allow us to address the paradox raised by [36]: on the one hand, several studies highlight the strong importance of shade trees for the sustainability of the production system, but on the other hand, farmers tend to remove shade trees to improve yields and do not notice any major drawback.

With respect to scaling-up, several studies have emphasized the importance of the landscape scale when considering ecological dynamics of agroecosystems [37]. Especially, the spatial distribution of insects involved in ecosystem services such as pest control has a strong effect on pest populations [38], [39]. In our study area, parasitism rate, for instance, has been shown to depend strongly on the distance to forest [40] and pollination also depends strongly on landscape structure [41]. Transferring our model to the landscape scale would make it possible to consider both ecological and economic interactions between different fields. Refining the management component of our model and transferring it to greater spatial scales would increase the number of tools available to balance between ecosystem services and disservices and give a better understanding of how ecological intensification could be put into practice.

## Conclusion

We modeled a Cacao agroecosystem under two management scenarios. The Cacao agroecosystem managed in an ecologically intensive way strongly differed from the one managed in a more conventional way using high quantities of chemical inputs. The ecologically intensive Cacao agroecosystem was more robust to pest outbreaks. It also showed higher yields when broad spectrum pesticides were used. However, the ecologically intensive Cacao agroecosystem was more sensitive to management-related perturbations and Cacao production perturbations, which confirms the high level of expertise needed to conduct such a management.

## Supporting Information

Appendix S1 **Description of the model.**
(DOCX)
