# Supplementary material for: Production and Robustness of a Cacao Agroecosystem: Effects of Two Contrasting Types of Management Strategies
Source: PLoS One. 2013 Dec 2;8(12):e80352. doi: 10.1371/journal.pone.0080352 (PMC3846552; doi:10.1371/journal.pone.0080352)
Supplement: Appendix S1 — Description of the model. (DOCX) [file pone.0080352.s001.docx]

*Production and robustness of a Cacao agroecosystem: effects of two contrasting types of management strategies.*

Rodolphe Sabatier, Kerstin Wiegand, Katrin Meyer, contact: [rodolphe.sabatier@agroparistech.fr](mailto:rodolphe.sabatier@agroparistech.fr)

**Appendix S1. Description of the model**

The model can be described by the following system:

$\left\{ \begin{matrix} {Pods}_{0} \left( t+1 \right)=f_{1}\left( t,N_{Pol}\left( t \right) \right) \\ Y\left( t \right)=f_{2}\left( {Pods}_{0}\left( t-5 \right),N_{CPB}\left( t-2 \right) \right) \\ \begin{matrix} N_{CPB}\left( t+1 \right)= f_{3}\left( {Pods}_{0}\left( t-3 \right),N_{CPB}\left( t \right),N_{Par}\left( t \right), \eta, \mathbb{1}_{\left\{ Spray \right\}}\left( t \right) \right) \\ N_{Par}\left( t+1 \right)=f_{4}\left( N_{Par}\left( t \right),N_{CPB}\left( t \right), \eta,\theta, \mathbb{1}_{\left\{ Spray \right\}}\left( t \right) \right) \\ N_{Pol}\left( t+1 \right)=f_{5}\left( N_{Pol}\left( t \right), \eta,\theta, \mathbb{1}_{\left\{ Spray \right\}}\left( t \right) \right) \end{matrix} \end{matrix} \right.$

With *f1* detailed in *eqn S.1.1*, *f2* in *eqn* *S.1.11*, *f3* in *eqn S.1.8*, *f4* in *eqn S.1.9*, *f5* in *eqn S.1.6*

***Dynamics of cacao pods***

The number of pods of age 0 that will become mature, *Pods0(t),* is the product of a yearly cyclic dynamics (eqn S.1.2) with an age factor (eqn S.1.3) and a pollination factor (eqn S.1.4) and can be written as follows:

${Pods}_{0}\left( t \right)=A\left( t \right) Pol\left( \psi(N_{Pol}\left( t-1 \right)) \right) P_{0}\left( t \right)$ (eqn S.1.1)

where *t* is the time in months, *P_0_(t)* is the intra-annual dynamics of pod production, *A(t)* the age factor and *Pol(𝜓(N_Pol_(t-1)))* is the pollination factor (depending on the pollinator population size at the former time step i.e. when future pods are still flowers). 𝜓 is the percentage of pollinated flowers that depends on *N_Pol_*, the size of the pollinator population. The model does not explicitly include mechanisms of pod loss and directly simulates the number of mature pods (age 5). Therefore pods of age > 0 are not explicitly represented. The number of pods of age *i* is simply *Pods_0_(t-i)*.

Intra-annual production of potential mature cacao pods of age 0, *P_0_(t),* is cyclic. It is modeled by the following equation (adapted from [1]):


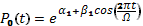
$P_{0}\left( t \right)=e^{\alpha_{1}+\beta_{1}cos\left( \frac{2\pi t}{\Omega} \right)}$ (eqn S.1.2)

where Ω is the time period of the sinusoid (=12 months). *α_1_* and β_1_ are the parameters of the sinusoid and were calibrated for the pod dynamics to reflect our study area (see the calibration section).

In order to reflect the inter-annual variation of cacao pod production, an age factor is added to this intra-annual dynamics of cacao pod production. This age factor *A(t)* is modeled using the following equation (adapted from [2]), reflecting a quite fast increase in cacao yield from 0 to 10 years followed by a slow decrease when cacao gets older:


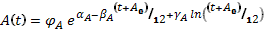
$A\left( t \right)={\varphi_{A}e}^{\alpha_{A}-\beta_{A} \left( t+A_{0} \right)/\Omega+\gamma_{A} ln\left( \left( t+A_{0} \right)/\Omega\right)}$ (eqn S.1.3)

where A_0_ is the initial average age of the cacao trees and α_A_, β_A_ and γ_A_ are constants that are calibrated to reflect our case study [2]. φ_A_ is a factor aimed at standardizing *A(t)*. It is calibrated to obtain *A(0)=1,* 120 months being the average age of Cacao trees found in our study area.

The number of potential mature pods of age 0 also depends on the intensity of pollination and this is included in the model by a pollination factor. The relationship between the number of pollinated flowers per tree 𝜓 (t) and the pollination factor *Pol(*𝜓(t*))* is adapted from [3]. This equation reflects a non linear increase of cacao yield with an increased proportion of pollinated flowers.

$Pol\left( \psi(t) \right)= \gamma_{P}\left( \alpha_{P}+\beta_{P}ln\left( \psi(t) \right) \right)$ (eqn S.1.4)

where α_P_ and β_P_ are the two pollination parameters of the equation developed by [3] and γ_P_ is the standardizing factor leading to *Pol(*𝜓(t*))=1* for the average pollination rate of the study area (10%; [3]). Given this low pollination rate, we do not expect any saturation effect and the number of pollinated flowers per tree, 𝜓 (t), is considered to be a linear function of the population size *N_Pol_(t)*.

$\psi\left( t \right)={\alpha_{\psi} N}_{Pol}\left( t \right)$ (eqn S.1.5)

***Ecological dynamics***

Three insect populations are modeled: the pollinator, the Cacao Pod Borer and its parasitoid. Dynamics are iterative, at each time step, insect populations may be impacted by the effects of spraying. We distinguish two effects of spraying: the efficiency *η* (the effect on the Cacao Pod Borer) and its selectivity θ (the ratio of effects on beneficial and on pest populations). Efficiency and selectivity range from 0 to 1, η *= 1* means that 100% of the Cacao Pod Borer are killed by the pesticides, θ *= 1* means that the effect of pesticide application on beneficial insects is as strong as its effect on Cacao Pod Borer.

*Pollinator dynamics*

Pollinator dynamics is iterative and population size was limited by a simple Beverton-Holt like density dependence function^[[1]](#footnote-1)^:

$N_{Pol}\left( t+1 \right)=\left( 1-\mathbb{1}_{\left\{ Spray \right\}}\left( t \right) \eta(1-\theta) \right) \frac{\lambda_{P}N_{Pol}\left( t \right)}{1+c_{P}N_{Pol}\left( t \right)}$ (eqn S.1.6)

where
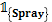
$\mathbb{1}_{\{Spray\}}$ is the characteristic function related to the spraying event. It is defined as follows:


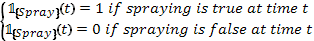
 $\left\{ \begin{matrix} \mathbb{1}_{\left\{ Spray \right\}}\left( t \right)=1 if spraying is true at time t \\ \mathbb{1}_{\left\{ Spray \right\}}\left( t \right)=0 if spraying is false at time t \end{matrix} \right.$ (eqn S.1.7)

𝜆_P_ is the population growth rate and *c_P_* the coefficient of density dependence.

*Dynamics of the Cacao Pod Borer and its parasitoid*

The Cacao Pod Borer and parasitoid dynamics were adapted from the work of [1]. In our model, we kept the same density dependence function and simplified the Cacao Pod Borer model to reduce the number of stages and modeled the parasitoid dynamics in an iterative way following the Nicholson and Bailey host-parasitoid model [4] to give memory to the parasitoid population.

The dynamics of the Cacao Pod Borer, *N_CPB_*(*t*+1), is modeled as follows:


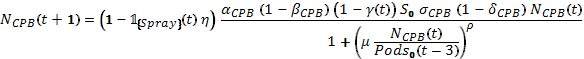
$N_{CPB}\left( t+1 \right)=\left( 1-\mathbb{1}_{\left\{ Spray \right\}}\left( t \right) \eta\right) \frac{\alpha_{CPB} \left( 1-\beta_{CPB} \right) {S_{0}\gamma\left( t \right) \sigma_{CPB} \left( 1-\delta_{CPB} \right) N}_{CPB}\left( t \right)}{1+\left( \mu\frac{N_{CPB}\left( t \right)}{{Pods}_{0}(t-3)} \right)^{\rho}}$ (eqn S.1.8)

where α_CPB_ is the number of eggs per female, *S_0_* the survival rate of larvae in the cacao pod, γ(t) is the survival rate of pupae due to parasitism, σ_CPB_ the sex ratio (0,5 for equal proportion of male and females), and 𝜇 and 𝜌 are the coefficients of density dependence. Predator populations (mainly birds and bats) are expected to be impacted by larger scale factors and predator dynamics were not included in the model. Predation was however included to the model through parameters 𝛿_CPB_, the predation rate of adults and β_CPB_, the predation rate of non-adults that mostly impacts mature larvae [5].

Most Cacao Pod Borer attacks occur on pods 3 months of age and we set the value of *S_0_* to the average larva survival rate in pods of this age [1]. Data from our study area confirmed that the sex ratio of the Cacao Pod Borer did not differ from 0.5 (Wielgoss and Clough unpublished data). Density dependence depends on the density of Cacao Pod Borers in pods of 3 months, therefore in *Pods_0_(t-3)*.

Parasitoid population dynamics, *N_Par_*(t+1), was modeled in an iterative, dynamic way. It was modeled through a Nicholson and Bailey host-parasitoid model [4] calibrated to fit the observations of Day (1985): parasitism rate between 0.5 and 0.05 per month.


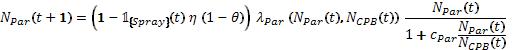
$N_{Par}\left( t+1 \right)=\left( 1-\mathbb{1}_{\left\{ Spray \right\}}\left( t \right) \eta\left( 1-\theta\right) \right) N_{CPB}\left( t \right)\left( 1-e^{-a_{Par} P\left( t \right)} \right)$ (eqn S.1.9)

where *a_Par_* is the probability that a given parasitoid will encounter a given host

Survival rate due to parasitism is then linked to *N_Par_* as follows:


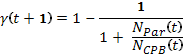
$\gamma\left( t \right)=e^{-a_{Par} N_{Par}\left( t \right)}$ (eqn S.1.10).

***Yield***

Yield *Y(t)* depends on the number of pods of age 5 months and on the yield loss due to the Cacao Pod Borer and is written as follows:


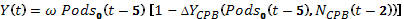
$Y\left( t \right)=\omega{Pods}_{0}\left( t-5 \right) \left[ 1-{\Delta Y}_{CPB}\left( {Pods}_{0}\left( t-5 \right),N_{CPB}\left( t-2 \right) \right) \right]$ (eqn S.1.11),

where 𝜔 is the average weight of dry bean per pod and *∆Y_CPB_* is the Yield loss due to the Cacao Pod Borer. *∆Y_CPB_* is adapted from [1] and reads as follows:


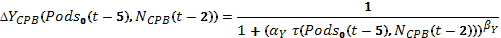
${\Delta Y}_{CPB}\left( {Pods}_{0}\left( t-5 \right),N_{CPB}\left( t-2 \right) \right)=\frac{1}{{1+\left( \alpha_{Y} \tau\left( {Pods}_{0}\left( t-5 \right),N_{CPB}\left( t-2 \right) \right) \right)}^{\beta_{Y}}}$ (eqn S.1.12),

where τ is the infestation rate of the cacao pods by the Cacao Pod Borer and α_Y_ and β_Y_ are the parameters of Cacao Pod Borer impact. *τ(Pods_0_(t-5),N_CPB_(t-2))* is defined as follows:


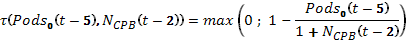
$\tau\left( {Pods}_{0}\left( t-5 \right),N_{CPB}\left( t-2 \right) \right)=max\left( 0 ; 1-\frac{{Pods}_{0}\left( t-5 \right)}{{1+N}_{CPB}\left( t-2 \right)} \right)$ (eqn S.1.13).

**References**

1. Day R (1985) Control of the Cocoa Pod Borer (Conopomorpha Cramerella) London: University of London.

2. Juhrbandt J (2011) Economic valuation of of land use change-A case study on rainforest conversion and agroforestry intensification in Central Sulawesi, Indonesia. Available: https://ediss.uni-goettingen.de/handle/11858/00-1735-0000-0006-AB32-C. Accessed 7 August 2013.

3. Groeneveld JH, Tscharntke T, Moser G, Clough Y (2010) Experimental evidence for stronger cacao yield limitation by pollination than by plant resources. Perspect Plant Ecol Evol Syst 12: 183–191. doi:10.1016/j.ppees.2010.02.005.

4. Hassell MP (1978) The spatial and temporal dynamics of host-parasitoid interactions. Oxford; New York: Oxford University Press.

5. Ooi PAC (1992) Prospects for biological control of cocoa insect pests. Cocoa pest and disease management in South east Asia and Australasia. pp. 101–107.

1. A Beverton-Holt function was preferred to a Ricker function since it avoided additional oscillations [↑](#footnote-ref-1)
